# Biosynthesized Selenium Nanoparticles Using Epigallocatechin Gallate Protect against Pentylenetetrazole-Induced Acute Epileptic Seizures in Mice via Antioxidative, Anti-Inflammatory, and Anti-Apoptotic Activities

**DOI:** 10.3390/biomedicines11071955

**Published:** 2023-07-11

**Authors:** Barakat M. Alrashdi, Alaa Fehaid, Rami B. Kassab, Sara Rizk, Ola A. Habotta, Ahmed E. Abdel Moneim

**Affiliations:** 1 Biology Department, College of Science, Jouf University, Sakaka 41412, Saudi Arabia; 2 Department of Forensic Medicine and Toxicology, Faculty of Veterinary Medicine, Mansoura University, Mansoura 35516, Egypt; 3 Department of Biology, Faculty of Science and Arts, Al-Baha University, Al-Baha 65799, Saudi Arabia; 4 Department of Immunizations and Vaccines, Hadayek Helwan Medical Center for Family Health, Cairo 4042342, Egypt; 5 Zoology and Entomology Department, Faculty of Science, Helwan University, Cairo 11792, Egypt

**Keywords:** epigallocatechin gallate, selenium nanoparticles, epilepsy, oxidative stress, inflammation, apoptosis, mice

## Abstract

Several negative outcomes are associated with current anti-epileptic medications. Epigallocatechin gallate (EGCG) is a plant-derived compound called catechin and has many medicinal activities, such as anti-inflammatory and antioxidant activities. Biosynthesized selenium nanoparticles are also showing their neuroprotective effect. The anti-epileptic effect of EGCG, alone or with SeNPs, is still debated. Here, we aimed to investigate the potential anti-seizure effect of biosynthesized SeNPs using EGCG (EGCG-SeNPs) against epileptic seizures and hippocampal damage, which is enhanced by pentylenetetrazole (PTZ) injection in mice. Mice were grouped as follows: control; PTZ-exposed group (epileptic model); EGCG + PTZ-treated group; sodium selenite (Na_2_SeO_3_) + PTZ-treated group; EGCG-SeNPs + PTZ-treated group; and valproic acid (VPA) + PTZ-treated group. EGCG-SeNPs administration showed anti-epileptic activity by increasing the latency time and reducing the seizure duration following the PTZ injection. Additionally, EGCG-SeNPs counteracted the PTZ-induced changes in oxidants and antioxidants. Moreover, EGCG-SeNPs inhibited the inflammatory response by suppressing the release of pro-inflammatory cytokines and decreasing the immunoreactivity of the glial fibrillary acidic protein and mRNA expression of glutamate receptor subunit zeta-1 (NMDAR; Grin1), showing their inhibitory effect on epilepsy-associated inflammation. Moreover, EGCG-SeNPs reduced PTZ-induced neuronal apoptosis, as indicated by a reduction in the levels of pro-apoptotic proteins and an elevation of the anti-apoptotic protein. Moreover, EGCG-SeNPs administration significantly modulated the PTZ-induced changes in monoamine levels and acetylcholinesterase activity in the hippocampal tissue. The obtained findings suggest the anti-seizure activity of EGCG-SeNPs via their antioxidant, anti-inflammatory, and anti-apoptotic effects, along with their neuromodulatory effect.

## 1. Introduction

Epilepsy is an enduring predisposition of the brain that induces seizures with abnormal neurobiological and psychological activities [1]. Epilepsy affects millions of people worldwide, resulting in many disabilities and mortalities for unclear reasons [2]. Epileptic degrees and existing symptoms are directly affected by the affected areas in the brain. It is well known that the most epileptogenic regions of the brain are the hippocampus and cerebral cortex [3]. Seizures are the most common sign of epileptic neurodegeneration [4]. Epileptic seizures have also been reported in cases of central nervous system (CNS) tumors, trauma, CNS developmental abnormalities, and inflammation [5].

The detailed mechanism of epileptic seizures is yet to be defined. However, the excitation and inhibition of neurons can control the neuronal response and epileptogenesis [6]. Moreover, oxidative stress, inflammation, apoptosis, and injury to neurons can induce epileptic seizures [7]. Oxidative damage is caused by an imbalance between prooxidants and antioxidants [8]. Brain tissues are susceptible to oxidative damage because of the high numbers of mitochondria, the high need for oxygen, and the low content of antioxidants [9]. In epilepsy, the sensitive nature of brain tissue increases neuronal oxidative damage, which is represented by elevated reactive oxygen (ROS) and nitrogen (RNS) species with reduced antioxidants [10]. Moreover, the over-release of ROS enhances neuronal hyperexcitability by altering the membrane potential of neurons, resulting in neuronal death either by necrosis or apoptosis [11].

Nuclear factor erythroid 2-related factor 2 (Nfe2L2) is a transcription factor that regulates the expression of antioxidant molecules, including superoxide dismutase (SOD), glutathione peroxidase (GPx), heme oxygenase-1 (HO-1), and glutathione (GSH) [8]. Due to the crucial role of Nfe2L2 in enhancing the antioxidative mechanism, many Nfe2L2-activating compounds have been investigated as therapeutic agents against oxidative damage-related diseases [12]. In addition, the excessive release of pro-inflammatory mediators, such as interleukins, tumor necrosis factor-alpha (TNF-α), and nuclear factor kappa B (NF-κB), was found to enhance neuronal hyperexcitability and epileptic seizures [13].

Epileptic seizures could be managed by the effects of anti-epileptic drugs (AEDs), including phenobarbital, valproic acid, topiramate, and carbamazepine. Depending on the seizure type, different mechanisms of action were employed, such as affecting voltage-dependent ion channels, modulating the neurotransmitters, enhancing Gamma-aminobutyric acid (GABA), and blocking glutamate [14]. The guidelines of the International League Against Epilepsy (ILAE) classified epileptic seizures into four types (focal, generalized, unknown, and unclassified) according to the onset of seizures [15]. Moreover, seizures were classified according to the awareness degree and the motor properties, which affect the choice of AEDs [14]. Up to 30% of epilepsy patients discontinue the use of the prescribed AED because of intolerance and adverse effects [16]. Negative effects of AEDs include hepatic toxicity, teratogenic effects, gastrointestinal disturbance, and continued seizures in the case of drug-resistant epilepsy [17,18].

Therefore, developing a novel anti-epileptic drug with minimal or no side effects is very important. Nowadays, many new therapies for different diseases are developed using nanomaterials that exhibit both efficiency and safety [19]. Nanoparticles have specific properties with a high surface area, and their uptake by different cells is directly related to their physicochemical properties, such as size, coating, and surface charge [20]. Among all, metallic nanoparticles have been reported to have high efficiency in treating neurodegenerative diseases [21]. Selenium nanoparticles (SeNPs) are widely used in the biomedical field as an anticancer, antifungal, and antimicrobial agent because of their higher bioactivity and lower cytotoxicity [22]. Many studies that investigated anti-epileptic therapies concluded the antioxidation, anti-inflammation, anti-apoptosis, and neuromodulatory activities of SeNPs [23,24]. In order to increase the biocompatibility of SeNPs, plant-based SeNPs were synthesized instead of chemically synthesized SeNPs [25]. Extracts of different herbs were successfully used in SeNP synthesis, such as hawthorn (berries), *Allium sativum* (garlic), and *Carica papaya* latex [26,27,28,29]. Plant-based SeNPs showed protective effects against damage to hepatic, renal, and neuronal tissues [24,30].

Epigallocatechin gallate (EGCG) is a plant-derived compound called catechin that positively impacts health. It is the major active compound in green tea and also exists in smaller quantities in berries, kiwis, apples, pistachios, and avocados [31]. EGCG acts as an antioxidant and protects the cells from free radical-induced damage [32]. Moreover, EGCG could induce an anti-inflammatory effect and treat neurodegenerative diseases such as Alzheimer’s and Parkinson’s [33].

In this study, we hypothesized that the synthesis of SeNPs using EGCG would exhibit more potent antioxidative and anti-inflammatory activities and treat epilepsy. Therefore, the anti-epileptic effect of EGCG delivered alone or with SeNPs will be investigated in detail. Finally, the current study aimed to study the potential anti-epileptic effect of biosynthesized SeNPs using EGCG against the epileptic seizures induced by pentylenetetrazole (PTZ) injection in mice and its mechanism.

## 2. Materials and Methods

### 2.1. Drugs

Epigallocatechin gallate (EGCG; Cas Number: 989-51-5) and pentylenetetrazole (PTZ; Cas Number: 54-95-5) were obtained from Sigma Chemical Co. (St. Louis, MO, USA).

#### Preparation and Characterization of EGCG-SeNPs

Briefly, 10 mL of sodium selenite (Na_2_SeO_3_, 10 mM) was mixed with 10 mL of EGCG (3.5 mg/mL) using magnetic stirring overnight. The developed solution (EGCG-SeNPs) was lyophilized by a vacuum freeze dryer (Labconco Freezone 4.5 Liter Freeze Dry System, Marshall Scientific, Hampton, NH, USA), and the developed powder was applied in the current experiment.

A zeta sizer analyzer (Zetasizer Nano ZS90, Malvern Panalytical, UK) was applied to determine the mean size and the surface charges of the EGCG-SeNPs. Additionally, transmission electron micrographs were obtained using a high-resolution transmission electron microscope (TEM; JEOL Ltd., Mitaka, Tokyo, Japan).

### 2.2. Experimental Design

Forty-eight male albino Swiss mice weighing 30–35 g and aged 5–6 weeks were obtained from VACSERA, Cairo, Egypt. Mice were housed under controlled laboratory conditions (12 h light/dark cycle; 25 ± 2 °C). Food and water were provided ad libitum. Before the experiment, mice were allowed to acclimatize to the laboratory environment for one week. The experimental protocol was approved by the Committee of Research Ethics for Animal Care, Helwan University (approval no. HU-IACUC/AEB0921-1).

Mice were randomly divided into six groups (*n* = 8 per group) and received different treatments as follows:

Group 1, CNTR group: Mice received physiological saline orally for two weeks. On the 14th day, mice were injected intraperitoneally (i.p.) with normal saline an hour following the oral administration of saline.

Group 2, PTZ-treated group (PTZ): mice received normal saline orally for two weeks. On the 14th day, mice received a single i.p. injection of PTZ (60 mg/kg, [34]) an hour following the oral administration of saline, as reported previously.

Group 3, ECGC + PTZ-treated group (ECGC + PTZ): Mice received a daily dose of ECGC (50 mg/kg orally) for two weeks, as reported previously [35]. On the 14th day, mice received a single i.p. injection of PTZ (60 mg/kg) an hour following the oral administration of ECGC.

Group 4, Na_2_SeO_3_ + PTZ-treated group (Na_2_SeO_3_ + PTZ): Mice received a daily dose of Na_2_SeO_3_ (0.5 mg/kg, orally) for two weeks, as previously reported [36]. On the 14th day, mice received a single i.p. injection of PTZ (60 mg/kg) an hour following the oral administration of Na_2_SeO_3_.

Group 5, EGCG-SeNPs + PTZ-treated group (EGCG-SeNPs + PTZ): Mice received a daily dose of ECGC-SeNPs (0.5 mg/kg, orally) for two weeks, as reported previously [37]. On the 14th day, mice received a single i.p. injection of PTZ (60 mg/kg) an hour following the oral administration of EGCG-SeNPs.

Group 6, valproic acid + PTZ-treated group (VPA + PTZ): Mice received a daily dose of sodium valproate (600 mg/kg, orally) for 14 days, as reported previously [38]. On the 14th day, mice received a single i.p. injection of PTZ (60 mg/kg) an hour following valproic acid administration.

Sterile saline was used for dissolving Na_2_SeO_3_, ECGC-SeNPs, and valproic acid for oral administration. One day after the last injection, mice were euthanized and sacrificed.

### 2.3. Seizures Induction by PTZ

PTZ acute injection was used to induce epileptic seizures in mice. Animals were observed for almost 45 min after PTZ injection, and seizure scores were recorded following the modified Racine scale [39] as follows:

State 0: no response; State 1: ear and facial twitching; state 2: myoclonic jerks; state 3: myoclonic jerks, rearing; state 4: turning over onto the side position, tonic-clonic seizures; state 5: turning over onto the back position, generalized tonic-clonic seizures. Moreover, the latency and duration of seizures were recorded.

### 2.4. Tissue Sampling

The hippocampus was immediately dissected, rinsed with physiological saline, and divided into small parts. To obtain a 10% (*w*/*v*) homogenate for biochemical examination, parts of hippocampal tissues were homogenized in 10 mM phosphate buffer (pH 7.4). For neurotransmitter evaluation, other parts of hippocampal tissues were blended in HPLC-grade methanol (75%, 10% *w*/*v*) and centrifuged for 12 min at 4500 rpm. The supernatant was exposed to HPLC. For histopathological studies, hippocampal tissues were fixed in 10% formalin, and the CA1 hippocampal region was examined microscopically for histopathological changes.

### 2.5. Estimation of Oxidant/Antioxidant Condition

To evaluate the oxidative stress in hippocampal tissues, lipid peroxidation (LPO) was estimated [40]. Nitric oxide (NO) levels were measured at 540 nm [41]. Moreover, Ellman’s method was used to analyze the reduced glutathione (GSH) levels [42]. The Abcam ELISA kit (ab201734) was employed to measure 8-hydroxy-2-deoxyguanosine (8OHdG) as an oxidative biomarker following the manufacturer’s instructions [43].

On the other hand, antioxidant capacity should be evaluated to judge the oxidant/antioxidant balance in the hippocampal tissues. Activities of superoxide dismutase (SOD), catalase (CAT), glutathione peroxidase (GPx), and glutathione reductase (GR) were measured using previously described techniques of Misra and Fridovich [44], Aebi [45], Paglia et al. [46], and Factor et al. [47], respectively.

### 2.6. Estimation of Inflammatory Biomarkers

The levels of tumor necrosis factor-α (TNF-α), and interleukin-1β (IL-1β) were determined with ELISA kits obtained from Cusabio Technology CO. (Houston, TX, USA) following the manufacturer’s instructions. 

### 2.7. Estimation of Apoptosis Biomarkers

The protein levels of Bcl-2 (B-cell lymphoma 2) and Bax (BCL-2-associated X protein) were measured in homogenates of cerebral hippocampal tissues using ELISA kits (CUSABIO Technology CO.), following the manufacturer’s instructions. Meanwhile, caspase-3 activity was measured using kits from Sigma-Aldrich (St. Louis, MO, USA) that depend on the colorimetric method following the manufacturer’s protocol.

### 2.8. Gene Expression Analysis

Total RNA samples were extracted from hippocampal tissues using the RNeasy Plus Mini-kit, and cDNAs were created using reverse transcriptase. Reverse transcription-quantitative polymerase chain reaction (RT-PCR) was performed on an Applied Biosystems 7500 using Power SYBR^®^ Green (Thermo Fisher Scientific, Waltham, MA, USA), and β-actin was used as a reference gene. PCR analysis condition was set as follows: 95 °C for 4 min, followed by 40 cycles of 10 s at 95 °C, 30 s at 60 °C, and 10 s at 72 °C. Data of gene expressions were shown as relative fold changes compared to the control’s expressions.

Table 1 shows the primer sequences of nuclear factor erythroid 2-related factor 2 (Nrf2; Nfe212), nitric oxide synthase 2 (Nos2), glutamate receptor subunit zeta-1 (NMDAR; Grin1), which encodes the expression of glutamate [NMDA] receptor subunit zeta-1, and the housekeeping (β-actin) genes.

### 2.9. Estimation of Neurotransmitters Levels

Levels of hippocampal 5-hydroxytryptamine (5-HT), dopamine (DA), and norepinephrine (NE) were analyzed by high-performance liquid chromatography (HPLC) according to the protocol described by Pagel et al. [48]. In addition, levels of gamma-aminobutyric acid (GABA) were measured according to the HPLC method described by Henrikson and Meredith [49]. Additionally, acetylcholinesterase (AChE) activity in the hippocampal tissue was determined using a colorimetric assay by Ellman et al. [50].

### 2.10. Estimation of Brain Astrocytes and Microglia Biomarkers

To estimate the glial fibrillary acidic protein (GFAP) as a main marker for brain astrocyte activation, GFAP was determined in the hippocampal tissues using an ELISA kit obtained from Merck (Cat. No. NS830, Darmstadt, Germany) following the supplier’s instructions. Brain-Derived Neurotrophic Factor (BDNF) was analyzed using an ELISA kit (Cat. No: MBS355435, MyBioSource, San Diego, CA, USA) according to the supplier’s instructions.

### 2.11. Immunohistochemistry Analysis

To evaluate the immunoreactivity of GFAP in the hippocampal tissue, sections (4–5 μm thickness) were incubated with anti-GFAP rabbit polyclonal antibodies overnight at 4 °C. Next, they were rinsed with PBS and incubated with biotinylated goat anti-rabbit immunoglobulins, followed by incubation with streptavidin-peroxidase complexes at 30 °C for 30 min. The peroxidase activity was determined using 3,3′-diaminobenzidine (DAB). Images were captured with an original magnification of ×400.

### 2.12. Histopathological Examination

Hippocampal tissues were dried and paraffinized at room temperature after fixation in 10% neutral buffered formalin for 24 h. The tissue was then sectioned into 4–5 µm thick sections, which were stained with hematoxylin and eosin (H&E) and examined under the microscope.

### 2.13. Statistical Analysis

The SPSS software version 20 application was used to statistically evaluate all of the data. The mean and standard deviation (SD) of the data were calculated. The difference between the mean values of distinct groups was calculated using a one-way ANOVA with Duncan multiple comparison tests. Statistical significance was defined as a *p*-value of less than 0.05.

## 3. Results

### 3.1. EGCG-SeNPs Characterization

**Figure 1:**
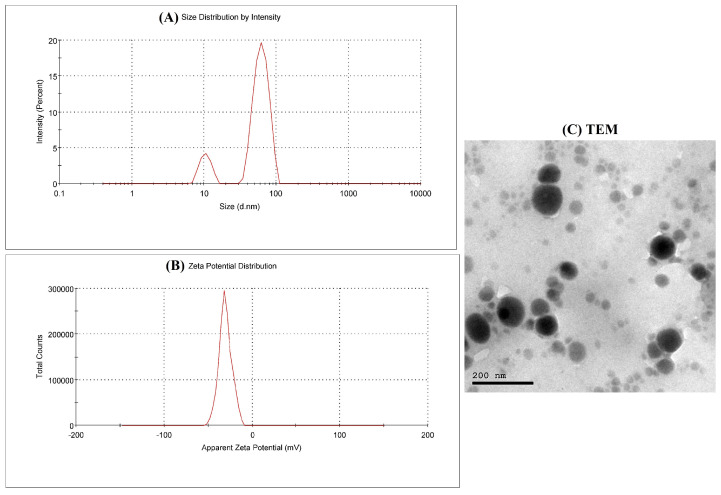
The characterization of the developed nanoformulation EGCG-SeNPs. (**A**) zeta sizer; (**B**) zeta potential and (**C**) TEM.

After mixing ECGC with Na_2_SeO_3_, a red color developed after being colorless. SeNPs were characterized by an average diameter of 89.3 nm and a mean zeta potential of −30.6 mV (Figure 1A,B). Additionally, transmission electron microscopy (TEM) was used to estimate the shape of the ECGC-SeNPs nanoformulation. The TEM photograph of ECGC-SeNPs showed spherical particles with a diameter of less than 100 nm (Figure 1C).

### 3.2. Epileptic Seizures Analysis

**Figure 2:**
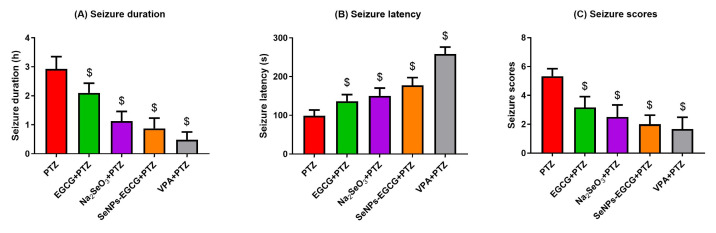
Effects of oral administration of epigallocatechin gallate (EGCG), sodium selenite (Na_2_SeO_3_), and biosynthesized SeNPs using EGCG (EGCG-SeNPs) on the seizure’s duration (**A**), latency (**B**), and scores (**C**) after the induction of epileptic seizures using pentylenetetrazol (PTZ). $ indicates a significant difference (*p* < 0.05) compared to the PTZ-exposed group. Data present the mean ± standard deviation (SD).

The chronic (for two weeks) pre-treatment of mice with EGCG, Na_2_SeO_3_, or EGCG-SeNPs and the acute injection of the anti-epileptic drug (valproic acid, VPA) could significantly (EGCG-SeNPs vs. PTZ; F = 42.70, *p* < 0.001) reduce the seizure duration (Figure 2). The latent period starting from the exposure time until the start of the seizures was significantly (EGCG-SeNPs vs. PTZ; F = 67.32, *p* < 0.001) longer in the treated groups when compared to the PTZ-exposed mice. In addition, the seizure scores were significantly (EGCG-SeNPs vs. PTZ; F = 28.80, *p* < 0.001) reduced in the treated groups when compared to the scores of the PTZ-exposed group. These data suggested the anti-convulsant effects of EGCG, Na_2_SeO_3_, and EGCG-SeNPs against PTZ-induced seizures.

### 3.3. Antioxidant Activity of EGCG-SeNPs against PTZ-Induced Oxidative Damage

**Figure 3:**
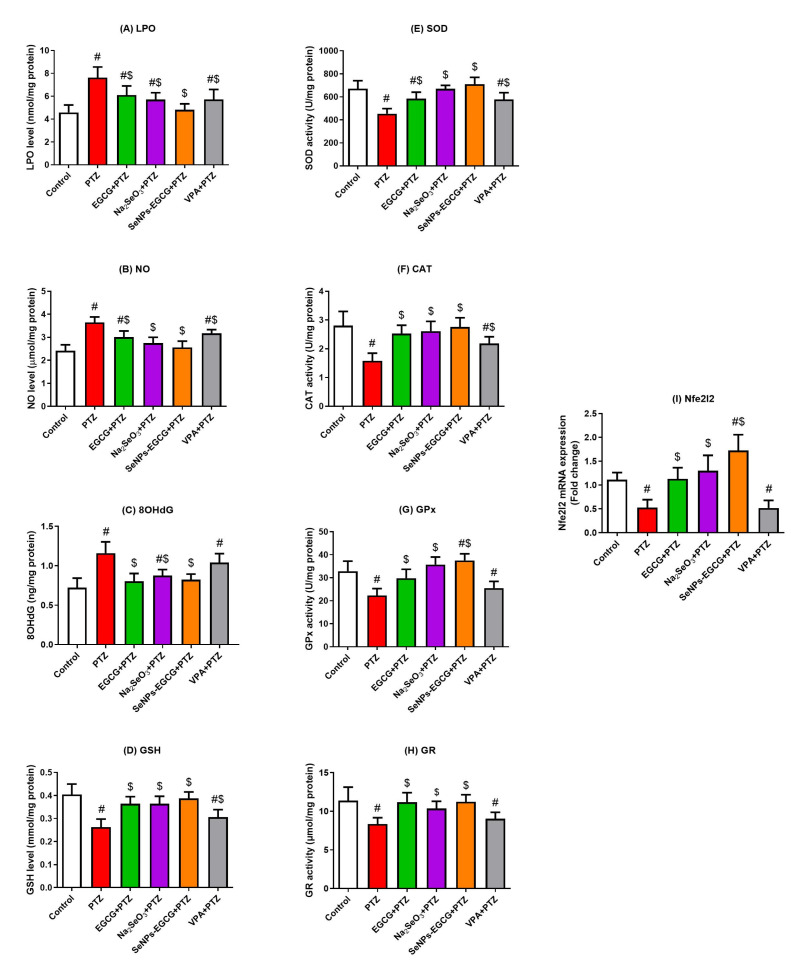
Antioxidant activities of oral administration of epigallocatechin gallate (EGCG), sodium selenite (Na_2_SeO_3_), and biosynthesized SeNPs using EGCG (EGCG-SeNPs) on PTZ-induced hippocampal oxidative damage. Levels of oxidants were indicated by lipid peroxidation (LPO, (**A**)), nitric oxide (NO, (**B**)), 8-hydroxy-2-deoxyguanosine (8OHdG, (**C**)), and glutathione (GSH, (**D**)). Antioxidants’ levels were indicated by superoxide dismutase (SOD, (**E**)), catalase (CAT, (**F**)), glutathione peroxidase (GPx, (**G**)), glutathione reductase (GR, (**H**)), and mRNA expression of the nuclear factor erythroid 2-related factor 2 (Nfe212, (**I**)). # and $ indicate significant differences (*p* < 0.05) compared to the control and PTZ-exposed groups, respectively. Data present the mean ± standard deviation (SD).

To evaluate the oxidant/antioxidant balance in the hippocampus, the levels of different oxidants and antioxidant molecules were measured in the hippocampal tissue. The exposure of mice to PTZ could enhance oxidative stress in the hippocampal tissues. This damage was confirmed by the increased levels of the oxidative biomarkers, including LPO, NO, and 8OHdG, along with the reduction in the GSH level as shown in Figure 3. In contrast, the antioxidant levels were reduced after PTZ exposure, including SOD, CAT, GPx, and GR enzymes, when compared to the control levels. Interestingly, the PTZ-induced changes in the oxidant/antioxidant status were significantly (EGCG-SeNPs vs. PTZ; F = 7.746 to 19.86, *p* < 0.015) restored to normal levels in the pre-treated groups with EGCG, Na_2_SeO_3_, and EGCG-SeNPs, as presented in Figure 3. VPA as a common anti-epileptic drug could also restore the oxidant/antioxidant balance, but not to the same positive significance (PVA vs. PTZ; *p* < 0.04 to >0.05) as the EGCG-SeNPs. The data shown suggest the potent antioxidant activity of EGCG-SeNPs against PTZ-induced oxidative damage. 

Nuclear factor erythroid-2-related factor 2 (Nfe212) modulates the antioxidant molecular activity in neuronal cells. Therefore, it was important to determine whether the Nfe212 had a role in the EGCG-SeNPs’ induced protective activities against the PTZ-induced neuro-damage or not by analyzing the Nfe212 mRNA expressions in the homogenates of the hippocampal tissues. As shown in Figure 3, the exposure of mice to PTZ downregulated Nfe212 mRNA expression compared to the control group. All the treatments could restore Nfe212 expression to normal ranges, with the highest levels in the EGCG-SeNPs-treated group (EGCG-SeNPs vs. PTZ; F = 19.21, *p* < 0.001). The data shown suggest the vital role of Nfe212 in the protective activities of EGCG-SeNPs against PTZ-induced hippocampal damage.

### 3.4. Anti-Inflammation Effect of EGCG-SeNPs against the PTZ-Induced Neuro-Inflammation

**Figure 4:**
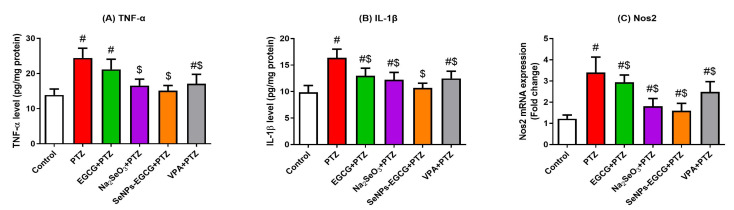
Anti-inflammatory activities of oral administration of epigallocatechin gallate (EGCG), sodium selenite (Na_2_SeO_3_), and biosynthesized SeNPs using EGCG (EGCG-SeNPs) on the PTZ-induced neuro-inflammation. Protein levels of tumor necrosis factor-α (TNF-α; (**A**)), interleukin-1β (IL-1β; (**B**)), and mRNA expression of NOS2 (**C**) were assessed as inflammation enhancers. # and $ indicate significant differences (*p* < 0.05) compared to the control and PTZ-exposed groups, respectively. Data present the mean ± standard deviation (SD).

Tumor necrosis factor-α (TNF-α), interleukin-1β (IL-1β), and nitric oxide synthase 2 (NOS2) enhance the inflammatory response in the brain tissues. The exposure of mice to PTZ enhanced (PTZ vs. Control; *p* < 0.0001) the protein levels of TNF-α and IL-1β, as well as the mRNA expression of NOS2 in the hippocampal tissue in comparison to the control group (Figure 4). While the EGCG-SeNPs-treated group showed the most significant (EGCG-SeNPs vs. PTZ; F = 16.89 to 18.29, *p* < 0.0001) restoration of TNF-α, IL-1β, and NOS2 levels when compared to the control and the PTZ-exposed groups. These data suggest the anti-inflammatory effect of EGCG-SeNPs against PTZ-induced neuro-inflammation.

### 3.5. Anti-Apoptotic Activity of EGCG-SeNPs against the PTZ-Provoked Neuronal Apoptosis

**Figure 5:**
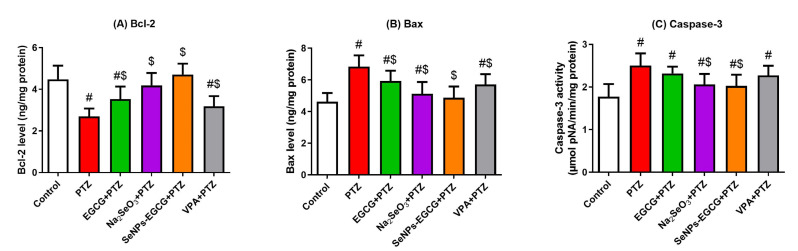
Anti-apoptotic activities of oral administration of epigallocatechin gallate (EGCG), sodium selenite (Na_2_SeO_3_), and biosynthesized SeNPs using EGCG (EGCG-SeNPs) on PTZ-induced neuronal apoptosis. Hippocampal levels of anti-apoptotic (Bcl-2; (**A**)) and pro-apoptotic proteins [Bax (**B**) and caspase-3 (**C**)] were measured in all groups. # and $ indicate significant differences (*p* < 0.05) compared to the control and PTZ-exposed groups, respectively. Data present the mean ± standard deviation (SD).

Neuronal apoptosis was assessed by quantitative analysis of the anti-apoptotic (Bcl-2) and pro-apoptotic (Bax and caspase-3) proteins in the hippocampal homogenates. As shown in Figure 5, PTZ exposure enhanced the apoptotic response in the hippocampus, as indicated by the significant increase in Bax and caspase-3 levels, and the significant decrease in Bcl-2 levels when compared to the control group. On the other side, EGCG-SeNPs treatment could show the best (EGCG-SeNPs vs. PTZ; F = 6.347 to 8.805, *p* < 0.002) restoration of the apoptotic molecules’ levels to the normal ranges in comparison to the control group, as shown in Figure 5. These data suggest the anti-apoptotic activity of EGCG-SeNPs against PTZ-induced neuronal apoptosis.

### 3.6. Effect of EGCG-SeNPs Treatment on the Neurotransmission

Protein levels of 5-HT, DA, NE, GABA, and AChE activity were measured in the hippocampal tissues. The PTZ-exposed group showed significant reductions in the hippocampal concentrations of all measured neurotransmitters compared to the control group. All the treated groups could significantly restore the neurotransmitters’ levels to normal levels when compared to both control and PTZ-exposed groups, as presented in Table 2. Among the treatments used, EGCG-SeNPs could enhance the best restoration levels of both NE and GABA neurotransmitters. The data shown suggest that EGCG-SeNPs can modulate hippocampal neurotransmitters during epileptic seizures.

### 3.7. The Effect of EGCG-SeNPs on the Neurological Function during the Epileptic Seizures

**Figure 6:**
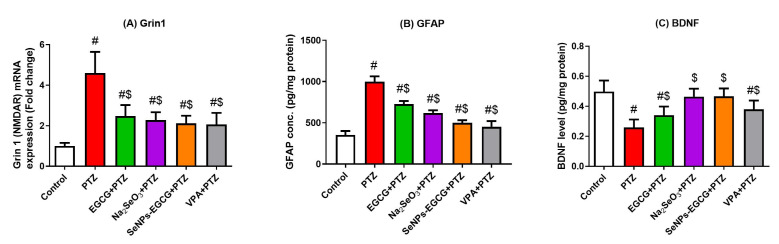
Effects of oral administration of epigallocatechin gallate (EGCG), sodium selenite (Na_2_SeO_3_), and biosynthesized SeNPs using EGCG (EGCG-SeNPs) on PTZ-induced neuronal dysfunction. mRNA expression of glutamate receptor subunit zeta-1 (NMDAR; (**A**)), protein levels of glial fibrillary acidic protein (GFAP; (**B**)), and brain-derived neurotrophic factor (BDNF; (**C**)) were measured in hippocampal tissue and compared to pentylenetetrazole (PTZ)-induced changes. # and $ indicate significant differences (*p* < 0.05) compared to the control and PTZ-exposed groups, respectively. Data present the mean ± standard deviation (SD).

**Figure 7:**
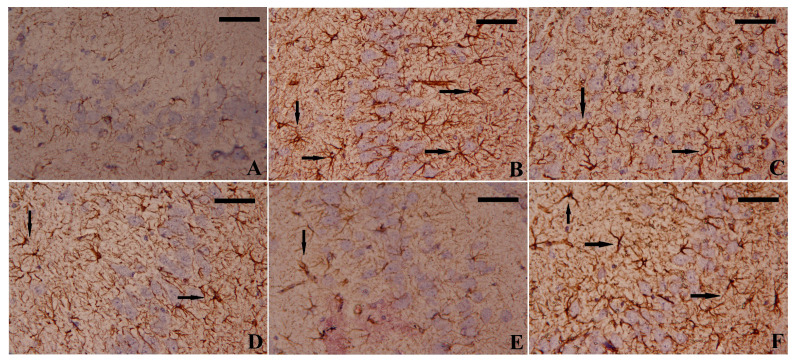
The immunoreactivity of glial fibrillary acidic protein (GFAP) in the hippocampal tissues in different treated groups. (**A**) Control group; (**B**): PTZ-treated group; (**C**) EGCG + PTZ-treated group; (**D**) Na_2_SeO_3_ + PTZ-treated group; (**E**) EGCG-SeNPs + PTZ-treated group; and (**F**) VPA + PTZ-treated group. magnifications = ×400. GFAPs are denoted by arrows; scale bar = 100 nm.

The mRNA expressions of glutamate receptor subunit zeta-1 (GRIN1), which encodes the expression of NMDAR, GFAP, and BDNF, were measured. The PTZ-exposed group showed a significant increase in both NMDAR mRNA expression and GFAP protein levels and a decrease in BDNF protein levels in the hippocampal tissues, indicating neuronal ion channel potentiation and astrocyte activation in this group (Figure 6). The upregulation of mRNA expression of GFAP was confirmed by immunohistochemistry analysis (Figure 7). However, the EGCG-SeNPs treatment could show significant (EGCG-SeNPs vs. PTZ; F = 14.63 to 20.84, *p* < 0.0001) restoration of NMDAR expression and both GFAP and BDNF levels when compared to the control and PTZ-exposed groups, as shown in Figure 6.

### 3.8. Effect of EGCG-SeNPs on the PTZ-Induced Histopathological Alterations during the Epileptic Seizures

**Figure 8:**
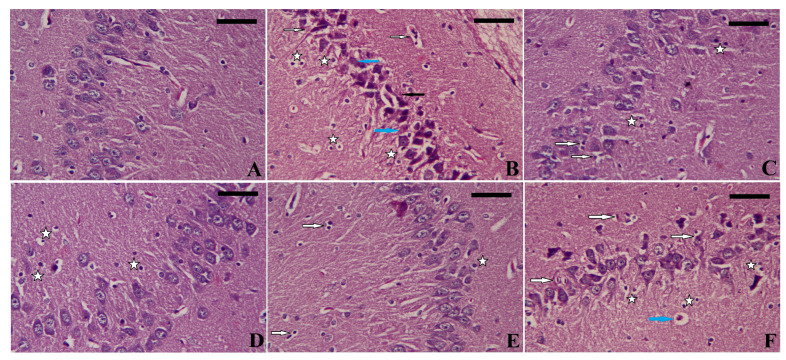
Effects of oral administration of epigallocatechin gallate (EGCG), sodium selenite (Na_2_SeO_3_), and biosynthesized SeNPs using EGCG (EGCG-SeNPs) on the PTZ-induced histopathological changes in the hippocampal tissue. (**A**) Control group; (**B**) PTZ-treated group; (**C**) EGCG + PTZ-treated group; (**D**) Na_2_SeO_3_ + PTZ-treated group; (**E**) EGCG-SeNPs + PTZ-treated group; and (**F**) VPA + PTZ-treated group. magnifications = ×400. White arrows indicate apoptotic neurons, white stars indicate pyknotic neurons, black arrows indicate necrotic neurons, and blue arrows indicate degenerated neurons. Scale bar = 100 nm.

The hippocampus architecture of the control rats was intact and healthy, but the PTZ-exposed animals exhibited degenerated, necrotic, and pyknotic neurons. On the other hand, as depicted in Figure 8, animals given ECGC-SeNPs displayed nearly normal histological architecture. However, inflammatory cell infiltration and apoptotic neurons were still seen in the sections.

## 4. Discussion

In the current research, we hypothesized that epileptic seizures might be treated by the protective activities of the biosynthesized EGCG-SeNPs. The basis of this hypothesis is that the SeNPs have already shown different therapeutic activities against many diseases when used either as primary or supplemental therapy, including neurodegenerative diseases such as Alzheimer’s disease, inflammatory conditions such as rheumatoid arthritis, and different cancer types [26,51]. SeNPs usually show high biocompatibility, stability, and cellular uptake ratios, which increase their current application, especially in the medical field [52]. Among the different methods of synthesizing SeNPs, green synthesis is considered the best to produce stabilized, environmentally friendly SeNPs with no toxic effects [53]. Therefore, epigallocatechin gallate, a plant-derived catechin, was chosen for the synthesis of SeNPs due to its antioxidant and anti-inflammatory properties, which might potentiate the protective effect of SeNPs against epileptic seizures in mice. Pentylenetetrazol injection was used to develop the hippocampal damage and the epileptic seizures in this study, and the PTZ-induced epilepsy was confirmed by the recorded seizure scores.

Due to the abundance of unsaturated fatty acids, high oxygen need, and low antioxidant activity, brain tissue is susceptible to oxidative injury progression [34]. Oxidative stress includes the imbalance between ROS production and antioxidant scavenging molecules and plays a critical role in epilepsy pathogenesis [54]. The effect of ROS-stimulated epilepsy was apparent in this study, as indicated by increased levels of oxidative markers (LPO, NO, GSH, and 8OHdG) and reduced levels of antioxidant markers (SOD, CAT, GR, and GPx) after PTZ injection. Lipoperoxidation takes place upon the interaction of hydroxyl radicals with unsaturated fatty acids in the neuronal membrane, which leads to the formation of lipid peroxide, hydroxide radicals, and MDA [55]. Following epileptic convulsions, there has been evidence of increased NO secretion. This increase could be linked to overexpression of the enzyme nitric oxide synthase (NOS), the rate-limiting enzyme in NO production. The negative effect of increased NO was caused by its interaction with superoxide anions, resulting in the formation of peroxynitrite radicals, which are associated with a variety of neurological effects [56]. The antioxidant system, including GSH, GPx, GR, SOD, and CAT, protected the cell from free radical accumulation [57]. The lessening of GSH and antioxidant activity has been linked to epileptic seizures in different models due to excessive ROS production, and this drop has been found to worsen with repetitive seizures [58,59].

Recently, the activation of the transcription factor Nrf2 has been targeted as a therapeutic strategy to reduce ROS-stimulated neuronal damage [60]. Nrf2 is a transcriptional factor that regulates the expression and function of cellular antioxidants (including GPx, GR, SOD, and CAT) to protect neurons from oxidative damage. Once deactivated, the antioxidant molecules are downregulated, as recorded in the current study.

Moreover, all the oxidative changes can disturb neurotransmitters, neuronal receptors, and enzymes’ normal functions, ending with neuronal cell damage and death [61]. As a result of the neuronal hyperexcitability and the ROS-induced neuronal damage, PTZ could activate caspase-3 and promote neuronal apoptosis, as indicated by the high levels of the pro-apoptotic marker (Bax) and the low levels of the anti-apoptotic marker (Bcl-2). ROS generation affects mitochondrial Ca^2+^ homeostasis, which impairs membrane permeability and the release of cytochrome c into the cytosol, resulting in the activation of caspase-9 and caspase-3, leading to the initiation of different cell death pathways [4]. In addition, ROS can mediate the inflammatory response via NF-κB signal transduction, which plays a clear role in epileptic seizures’ development [13]. IL-1β is a pro-inflammatory cytokine that promotes cyclooxygenase-2 activity to produce prostaglandin. Prostaglandin is a prostacyclin precursor that stimulates the astrocytes to produce glutamate, ending with seizures-associated neuronal hyperexcitability [62]. This explains the noted elevations of TNF-α and IL-1β levels, as well as the mRNA expression of NOS2 in the hippocampal tissues of mice after PTZ injection in this study.

Following the treatment of mice using the EGCG-SeNPs, all the oxidative, inflammatory, and apoptotic neuronal responses were reduced by the protective action of the EGCG-SeNPs in the current study. This could be explained by the specific properties of SeNPs, including their high surface area and nanosizing, which induce higher cellular uptake and free radical scavenging in the neuronal tissues [63]. SeNPs could upregulate Nfe212 mRNA expression and alleviate neuronal lipid peroxidation and dysfunction in the hippocampus in previous research as well [64]. Moreover, EGCG exhibited anti-inflammatory activity against the lipopolysaccharides (LPS)-treated cells via controlling the Nfe212 and HO-1 gene expressions [65]. ECGC could also induce apoptosis of different cancer cell lines, showing its potential anti-apoptotic effect [66]. Our findings suggest that EGCG and SeNPs can potentiate each other’s effects and induce their potent antioxidative, anti-inflammatory, and anti-apoptotic responses against PTZ-induced neuronal damage.

Our findings confirm that PTZ could induce astrocyte activation, which is indicated by the increased levels of GFAP. Also, the neuronal level of brain-derived neurotrophic factor (BDNF) was decreased after exposure of mice to PTZ, suggesting neuronal damage. The overproduction of ROS and pro-inflammatory cytokines is linked with the activation of astrocytes, which then enhance astrogliosis via the increased expression of GFAP [67]. This mechanism is significant in epileptic conditions and promotes epileptic seizures [68]. Many researchers have recently targeted the BDNF pathway to suppress epileptic seizures [69]. Since there is previous evidence supporting that glial cells can modulate the synaptic transmission between neural cells [70], different neurotransmitters were measured in this study, including 5-hydroxytryptamine (5-HT), dopamine (DA), norepinephrine (NE), gamma-aminobutyric acid (GABA), and acetylcholinesterase (AChE) activity in the hippocampal tissues. The reduction in neurotransmitter levels following PTZ-induced epilepsy is based on the augmented excitatory glutamate release, which is the central modulator in epileptic seizures [71]. Moreover, AChE alters neuronal excitability, promotes synaptic plasticity, and modulates memory and neuronal functions [72]. The deactivation of AChE is linked with the accumulation of acetylcholine at the synapse, resulting in the progression of convulsions [73]. Interestingly, it was evident that the treated mice with EGCG-SeNPs could restore all the changes in the neuronal biomarkers and neurotransmitter levels to their normal ranges compared to the control and PTZ-exposed groups. The provoked neuroprotective effect is directly related to the ability of EGCG to reduce neuronal oxidative damage and cell death. Previous research was in accordance with our data, concluding that green tea extract (which includes EGCG) supplementation can inhibit cognitive defects and oxidative stress in an Alzheimer’s disease murine model [74]. The neuroprotective effect is not limited to the action of EGCG but also extended to the nanoselenium role, which has been concluded previously, showing its ameliorative results against neurological diseases via their antioxidant and anti-inflammatory effects [23,58,75]. Altogether, ROS production and the resultant oxidative damage are one of the main modulators of epileptic seizures and neurodegenerative damage, which were enhanced by the PTZ exposure in our study. Treatment of mice with green synthesized SeNPs using EGCG could show a potent antioxidative activity that scavenges the released free radicals and consequently reduces the inflammatory and apoptotic responses. Moreover, neuronal function and transmission were also regulated by the effect of EGCG-SeNPs via their antioxidant activity, showing reduced epileptic seizures as scored and analyzed.

## 5. Conclusions

This research concluded that the biosynthesized selenium nanoparticles using epigallocatechin gallate (EGCG-SeNPs) have potential neuroprotective and anti-seizures activities against PTZ-induced epileptic seizures in mice due to their antioxidation, anti-inflammation, and anti-apoptosis activities, as well as their neurotransmitter regulation.

## Figures and Tables

**Table 1:** Primer sequences of genes analyzed by RT-qPCR.

Gene	Accession Number	Forward (5′–-3′)	Reverse (3′–-5′)
**Nrf2**	NM_001399173.1	CCTCAGCATGATGGACTTGGA	GCGACTGAAATGTAGGTGAAGA
**iNOS**	NM_012611.3	GGTGAGGGGACTGGACTTTTAG	TTGTTGGGCTGGGAATAGCA
**NMDAR**	NM_012573.4	GGCAACTTGTATGGGAGCCT	ATTTACCGCCTGTGATGGCA
**β-actin**	NM_031144.3	GTCCACCCGCGAGTACAAC	GGATGCCTCTCTTGCTCTGG

**Table 2:** Effects of oral administration of epigallocatechin gallate (EGCG), sodium selenite (Na_2_SeO_3_), and biosynthesized SeNPs using EGCG (EGCG-SeNPs) on monoamines [dopamine (DA), norepinephrine (NE), and serotonin (5-HT)] and gamma-aminobutyric acid (GABA) levels and acetilcolinesterase activity after the induction of epileptic seizures using pentylenetetrazol (PTZ).

Parameters	Control	PTZ	EGCG + PTZ	Na_2_SeO_3_ + PTZ	SeNPs + PTZ	VPA + PTZ
**5-HT (μg/g tissue)**	12.63 ± 1.27	6.39 ± 0.94 ^#^	8.61 ± 0.2.27 ^#$^	10.79 ± 1.56 ^#$^	11.93 ± 1.19 ^$^	13.25 ± 1.98 ^$^
**DA (μg/g tissue)**	0.42 ± 0.08	0.15 ± 0.02 ^#^	0.34 ± 0.09 ^#$^	0.38 ± 0.09 ^$^	0.43 ± 0.07 ^$^	0.49 ± 0.09 ^$^
**NE (μg/g tissue)**	0.55 ± 0.09	0.27 ± 0.04 ^#^	0.35 ± 0.08 ^#$^	0.46 ± 0.07 ^#$^	0.54 ± 0.08 ^$^	0.53 ± 0.09 ^$^
**GABA (μg/g tissue)**	884.0 ± 71.70	624.4 ± 109.5 ^#^	732.3 ± 210.8 ^$^	813.5 ± 143.9 ^$^	875.2 ± 72.4 ^$^	821.5 ± 139.3 ^$^
**AChE (µmol/min/mg protein)**	9.79 ± 1.83	5.08 ± 1.52 ^#^	6.16 ± 1.95 ^#$^	9.38 ± 1.84 ^$^	8.64 ± 1.27 ^#$^	8.97 ± 1.30 ^$^

# and $ indicate significant differences (*p* < 0.05) compared to the control and PTZ-exposed groups, respectively. Data present the mean ± standard deviation (SD).

## Data Availability

All relevant data are within the paper.
